# Network analysis identifies a putative role for the PPAR and type 1 interferon pathways in glucocorticoid actions in asthmatics

**DOI:** 10.1186/1755-8794-5-27

**Published:** 2012-06-19

**Authors:** Diego Diez, Susumu Goto, John V Fahy, David J Erle, Prescott G Woodruff, Åsa M Wheelock, Craig E Wheelock

**Affiliations:** 1 Bioinformatics Center, Institute for Chemical Research, Kyoto University, Uji, Kyoto, 611-0011, Japan; 2 Division of Pulmonary and Critical Care Medicine, Department of Medicine, University of California San Francisco, San Francisco, CA, USA; 3 Lung Biology Center, University of California San Francisco, San Francisco, CA, USA; 4 Cardiovascular Research Institute, University of California San Francisco, San Francisco, CA, USA; 5 Respiratory Medicine Unit, Department of Medicine, Karolinska Institutet, Stockholm, Sweden; 6 Department of Medical Biochemistry and Biophysics, Division of Physiological Chemistry II, Karolinska Institutet, Stockholm, Sweden; 7 Laboratory of Bioinformatics and Genomics, World Premier International Immunology Frontier Research Center, Osaka University, Osaka, 565-0871, Japan

**Keywords:** Asthma, Inflammation, Glucocorticoids, Fluticasone propionate, Flovent, Network analysis, PPAR pathway, Toll-like receptor pathway, Interferon pathway

## Abstract

**Background:**

Asthma is a chronic inflammatory airway disease influenced by genetic and environmental factors that affects ~300 million people worldwide, leading to ~250,000 deaths annually. Glucocorticoids (GCs) are well-known therapeutics that are used extensively to suppress airway inflammation in asthmatics. The airway epithelium plays an important role in the initiation and modulation of the inflammatory response. While the role of GCs in disease management is well understood, few studies have examined the holistic effects on the airway epithelium.

**Methods:**

Gene expression data were used to generate a co-transcriptional network, which was interrogated to identify modules of functionally related genes. In parallel, expression data were mapped to the human protein-protein interaction (PPI) network in order to identify modules with differentially expressed genes. A common pathways approach was applied to highlight genes and pathways functionally relevant and significantly altered following GC treatment.

**Results:**

Co-transcriptional network analysis identified pathways involved in inflammatory processes in the epithelium of asthmatics, including the Toll-like receptor (TLR) and PPAR signaling pathways. Analysis of the PPI network identified *RXRA*, *PPARGC1A*, *STAT1* and *IRF9*, among others genes, as differentially expressed. Common pathways analysis highlighted TLR and PPAR signaling pathways, providing a link between general inflammatory processes and the actions of GCs. Promoter analysis identified genes regulated by the glucocorticoid receptor (GCR) and PPAR pathways as well as highlighted the interferon pathway as a target of GCs.

**Conclusions:**

Network analyses identified known genes and pathways associated with inflammatory processes in the airway epithelium of asthmatics. This workflow illustrated a hypothesis generating experimental design that integrated multiple analysis methods to produce a weight-of-evidence based approach upon which future focused studies can be designed. In this case, results suggested a mechanism whereby GCs repress TLR-mediated interferon production via upregulation of the PPAR signaling pathway. These results highlight the role of interferons in asthma and their potential as targets of future therapeutic efforts.

## Background

Asthma is a chronic inflammatory airway disease influenced by genetic and environmental factors, characterized by variable and recurring symptoms (wheezing, coughing, chest tightness, shortness of breath), airflow obstruction and bronchospasm [1]. Asthma affects ~300 million people worldwide, leading to ~250,000 deaths annually [2]. The majority of the asthmatic population is characterized by the development of an inflammatory response following allergen exposure (allergic asthma). The airway epithelium plays a critical role in allergen sensitization in asthma. Allergens and allergen products activate specific receptors in the surface of the epithelial cells, including Toll-like receptors (TLRs). The activated epithelium releases inflammatory cytokines that enhance dendritic cell migration to lymph nodes, recruitment of innate immune and Th2 cells to the airways, and activation of Th2 cells [3,4].

While no cure exists to date, glucocorticoids (GCs) are commonly prescribed to control asthma symptoms [5,6]. Glucocorticoids bind to the glucocorticoid receptor (GCR), which interacts and represses (trans-repression) the activity of transcription factors that regulate the expression of inflammatory genes (*e.g.,* transcription factors AP1 and NFKB1). The activated GCR also binds to glucocorticoid response elements (GRE) in the DNA and promotes the expression of genes with anti-inflammatory actions (*e.g., GILZMKP-1*) [7].

Although the effects of GCs on the airway epithelium are well known, the mechanisms mediating GCR actions are not as well understood. A significant percentage of the asthmatic population requires the maximum doses of inhaled GCs and suffers the associated side effects and a small fraction of asthmatics are resistant to GCs [8]. Accordingly, increased insight into the molecular mechanisms mediating GC anti-inflammatory actions will assist in the development of therapies that minimize resistance and side effects as well as increase our understanding of the disease. In addition, an understanding of individual responses to GC treatment will assist in establishing treatment regimens tailored for personal susceptibility to GCs.

We previously investigated the effects of a synthetic GC, fluticasone propionate (Flovent), on the transcriptional profile of the airway epithelium of asthmatics [9]. In that study the aim was to identify genes regulated by GCs in the airways, and therefore the focus was on the most significantly altered genes. We demonstrated the GC-dependent regulation of a number of genes, including *CLCA1, POSTN, SERPINB2* and *FKBP5*[9]. To gain further insight into the processes governing the actions of GCs in the airways, we performed an unbiased analysis to interrogate the microarray dataset from a systems-based perspective. Our approach was primarily that of a hypothesis generating experiment, where two complementary network approaches were applied to mine different aspects of cellular networks. The first method used co-transcriptional information to characterize the functional pathways activated in the airways. The second method combined protein-protein interaction (PPI) networks with gene expression changes to identify pathways altered following Flovent treatment. Subsequently, combining results from both networks provided a weight of evidence approach to elucidate mechanistic processes in the action of Flovent in asthmatics. These results were further supported by promoter enrichment analysis to highlight a putative role of PPAR in suppressing the TLR-mediated interferon pathway following GC treatment. The combination of these different approaches demonstrated how multiple analysis methods can be combined in an *in silico* experiment to generate novel hypotheses. Taken together, these efforts provided a concerted investigation into the action of GCs in the airway epithelium of asthmatics.

## Results

### Statistical analysis

**Figure 1:**
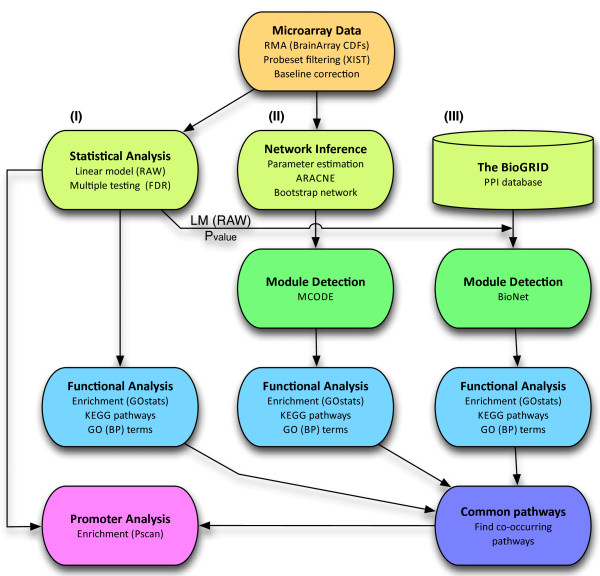
**Overview of the analysis workflow.** This figure shows an overview of the analysis workflow followed in this study as described in the text. From the initial microarray data (orange), three different approaches were pursued (light green). (**I**) Statistical analysis (**II**) Co-transcriptional network analysis and (**III**) Interactome network analysis. Each of these paths ends with a functional analysis (blue), which was combined into a common pathways analysis at the end of the pipeline (purple). Promoter analysis (lavender) was performed afterwards as a validation step for the conclusions from the common pathways analysis. LM = linear models. RAW = unadjusted *P*-values (not corrected for multiple hypothesis testing).

Figure 1 shows an overview of the analysis workflow followed in this study. Linear model analysis of differences due to Flovent treatment revealed 1,492 genes that were differentially expressed in Flovent versus placebo treated subjects (*P < 0.05*, Additional file 1: Table S1). The *P*-values were not corrected for multiple testing and therefore the list of genes is referred to as ‘RAW’. This list included many genes with a purported role in regulation of the immune response in asthma: interleukins (*IL2IL27* and *IL33*), chemokines (*CXCL1CXCL17CCL5* and *CCL27*), mucins (*MUC2MUC13* and *MUCL1*), *TLR7* and *NOS2*, among others. Functional enrichment analysis resulted in 13 significantly enriched KEGG pathways and 132 GO biological processes (*P < 0.05*) (Additional file 2: Table S2). Additional file 3: Figure S1 shows the 10 KEGG pathways that resulted after filtering (see Methods), including the top ones ‘Allograft rejection’, ‘Antigen processing and presentation’ and ‘Glutathione metabolism’. Results for GO enrichment included: ‘antigen processing and presentation of peptide antigen via MHC class I’, ‘antigen processing and presentation’ and ‘positive regulation of B cell proliferation’ as the top ranking biological processes. Although the list of uncorrected *P*-values (RAW) contained many genes and pathways related to asthma, the results possessed a potential high false positive rate (16,015 x 0.05 = 800 theoretical false positives). After correction for multiple testing by the methods of Benjamini and Hochberg [10], 115 genes remained significantly altered with q < 0.05 (Additional file 1: Table S1). This list of genes is referred to as ‘FDR’, and subsequent KEGG pathway enrichment resulted in five pathways overrepresented; ‘Steroid hormone biosynthesis’, ‘Sulfur metabolism’, ‘Primary bile acid biosynthesis’, ‘Endocytosis’ and ‘Retinol metabolism’ (Additional file 2: Table S2 and Additional file 3: Figure S1). GO enrichment analysis returned 123 significant terms including ‘negative regulation of B cell activation’ and ‘B cell mediated immunity’ and other biological processes related to asthma. However, only a few genes were associated with these selected terms (1–2 genes with *P* ~ 1E-03).

### Analysis of the co-expression network

**Figure 2:**
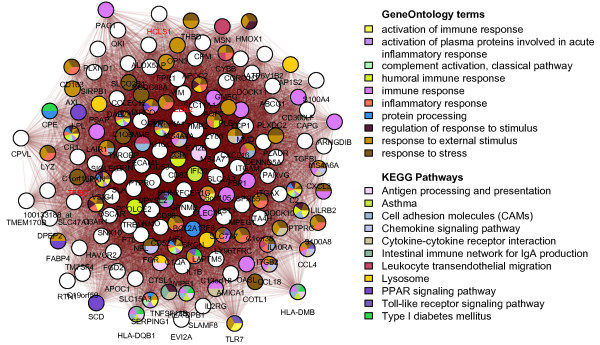
**MCODE module M1.** Selected KEGG and GO enriched terms in the MCODE module M1. This module contains the greatest number of genes of all modules identified via MCODE from the co-transcriptional network and has the highest MCODE score (*i.e.,* connectivity density). A red label indicates a gene annotated in GO (biological process) with transcription factor activity. This module contains no differentially expressed genes identified at *P < 0.05.*

A co-transcriptional network was constructed from the microarray data using the ARACNE method [11]. The network was built based upon within subject differences in transcript levels between the first sample (pre-treatment) and the second sample (after Flovent treatment or placebo). The inferred network contained 7,676 nodes and 49,499 edges (Additional file 3: Figure S2), and the degree distribution followed a power-law with γ = 1.69 (Additional file 3: Figure S3). The MCODE method was applied to identify clusters of genes with similar expression profiles in the inferred network [12]. This method was originally designed to detect complexes in PPI networks, but can also be used to detect clusters in co-transcriptional networks. MCODE retrieved 109 modules, with module 1 (M1) being the highest ranked (*i.e.,* greatest connectivity in the network). Module M1 contained 150 highly correlated genes (average *Spearman* ρ = 0.8074; Figure 2). Pathway analysis resulted in 19 significantly enriched KEGG pathways (*P < 0.05,* Additional file 2: Table S2), which were reduced to 11 after pathway filtering (Additional file 3: Figure S1). Most of the pathways enriched in M1 are directly related to biological processes in asthma, including ‘Asthma’, ‘Cell adhesion molecules (CAMs)’, ‘Antigen processing and presentation’, ‘Cytokine-cytokine receptor interaction’, ‘Toll-like receptor signaling pathway’, ‘PPAR signaling pathway’, ‘Leukocyte transendothelial migration’ and ‘Chemokine signaling pathway’. GO enrichment analysis revealed 313 significantly enriched biological processes (*P < 0.05*), with many categories related to asthma pathogenesis located top in the ranking (*i.e.,* had lowest *P*-values). In particular, the most significant term was ‘inflammatory response’ with 28 associated genes (*P* = 1.12x10^-19^), including chemokine ligands (*CXCL3CCL4CCL18*), chemokine receptor (*CCR1*), complement components (*C1QAC1QBC1QCC2*), integrins (*ITGB2*), interleukins (*IL1B*) and lymphocyte antigens (*LY86*). Other top-ranked terms included ‘leukocyte migration’, ‘regulation of T cell activation’, ‘neutrophil chemotaxis’ and ‘regulation of interleukin 2 production’ (Additional file 2: Table S2).

The remaining modules generally contained less information, with the exception of modules M5, M6 and M9. Module M5 contained the greatest number of differentially expressed genes (Additional file 3: Figure S4, 26 differentially expressed genes at *P < 0.05*, and 22 genes with q < 0.05), which were both negatively and positively correlated (absolute mean *Spearman* ρ = 0.665; Additional file 3: Figure S5). Only three KEGG pathways were overrepresented, including ‘Steroid hormone biosynthesis’ (Additional file 2: Table S2). GO functional analysis revealed 40 biological processes; however, 35 of them consisted of only a single gene association. Overrepresented terms included ‘steroid metabolic process’, ‘thiamin transport’, ‘positive regulation of epithelial cell proliferation’ and ‘progesterone receptor signaling pathway’ (Additional file 2: Table S2). Module M6 contained 39 correlated genes (average *Spearman* ρ = 0.6730; Additional file 3: Figures S5 and S6) and appeared to be functionally related to module M1. Some of the genes in module M6 participate in pathways enriched in module M1, including *TLR4* ('Toll-like receptor signaling pathway'), *PPARG* and *NFAM1* ('PPAR signaling pathway'). Module M9 had 7 correlated genes (average *Spearman* ρ = 0.8055) genes, including *JUN*, *JUNB* and *FOS*, which are part of the *AP1* transcription factor that regulates the expression of inflammatory genes, and is a target of the activated GCR. The enriched KEGG pathways that passed the filtering were ‘B-cell receptor signaling pathway’, ‘MAPK signaling pathway’, ‘T cell receptor signaling pathway’ and ‘Toll-like receptor signaling pathway’ (Additional file 3: Figure S7; Additional file 2: Table S2). The top-ten ranked GO terms included ‘response to cytokine stimulus’ and ‘SMAD protein signal transduction’ (Additional file 2: Table S2).

### Analysis of the human interactome

**Figure 3:**
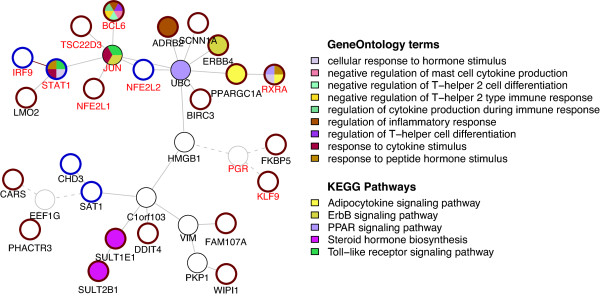
**BioNet module.** BioNet module annotated with selected KEGG pathways and GO terms obtained from functional enrichment analysis. Differentially expressed genes (*P < 0.05*) are indicated with a red circle (up-regulated in response to Flovent treatment) or a blue circle (down-regulated in response to Flovent treatment). A red label indicates a gene annotated in GO (biological process) with transcription factor activity. Grey circles indicate genes not expressed (based on *XIST* filtering - see Methods), which are connected to other genes through gray dashed lines.

Protein-protein interaction (PPI) networks represent the skeleton on which signaling pathways are constructed and can be used to identify modules of differentially expressed genes related to the same or overlapping signaling pathways [13]. The human interactome was obtained from the BioGRID database, and the BioNet software was used to identify modules significantly enriched in differentially expressed genes [14,15]. BioNet detected a subnetwork containing 32 genes, including 19 upregulated and 4 downregulated (*P < 0.05*, 19 with q < 0.05) by Flovent treatment (Figure 3). KEGG pathway enrichment analysis of this module highlighted 9 significant pathways including ‘Toll-like receptor signaling pathway’, ‘Steroid hormone biosynthesis’, ‘PPAR signaling pathway’, ‘ErbB signaling pathway’, and ‘Adipocytokine signaling pathway’ (Additional file 3: Figure S1; Additional file 2: Table S2). GO analysis identified 207 processes, with ‘alpha-beta T cell activation during immune response’ as the top-third term, although it only had a single associated gene. Some other significant biological processes not in the top rank have a direct relation to asthma pathogenesis, including ‘negative regulation of T-helper 2 type immune response’, ‘response to cytokine stimulus’, ‘regulation of inflammatory response’, ‘SMAD protein signaling transduction’ and ‘cellular response to hormone stimulus’ (Additional file 2: Table S2). However, these terms also show a low number (1–2) of associated genes.

### Analysis of common pathways

**Figure 4:**
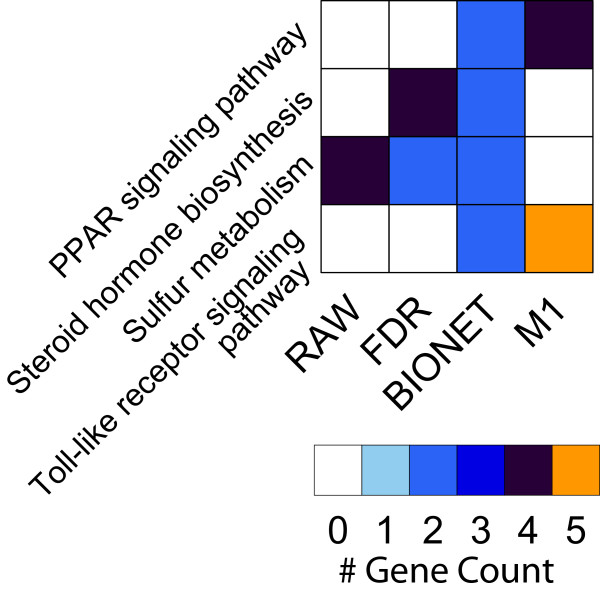
**Common pathways analysis.** Analysis of common pathways highlighted pathways present in the BioNet and selected MCODE modules. This approach resulted in Toll-like receptor signaling and PPAR signaling pathways as the pathways in common with other modules. See Additional file 3: Figure S1 for a full summary of the functional enrichment analysis.

Networks were constructed using the two different approaches of co-transcriptional and PPI-based analyses. To identify cellular processes active in the airways of asthmatics that are targeted by GC treatment, we examined common pathways found in the different modules. We focused on common pathways between the highest ranked MCODE module (M1) and the PPI module (BIONET). The pathways, along with the number of genes in each pathway, are shown in the heatmap in Figure 4. The only common pathways were ‘Toll-like receptor signaling pathway’ and ‘PPAR signaling pathway’. To determine whether these two pathways appeared in common by chance, we performed a simulation with random lists of genes of the same size as the BIONET and M1 modules. For each random list, we performed enrichment analysis and determined the common pathways. By repeating this process 1,000 times and computing the number of common pathways similar to those detected in our analysis of the Flovent dataset, we estimated the probability of observing common pathways by chance. The simulation resulted in a probability of random appearance as common pathways of *P = 0.002* for ‘Toll-like receptor signaling pathway’. ‘PPAR signaling pathway’ did not appear as a common pathway in any of the simulations (*P = 0*). These results demonstrate the robustness of the common pathways approach.

### Promoter enrichment analysis

The analysis of common pathways highlighted the TLR and PPAR pathways as targets of GC actions. Consequently, some of the genes regulated by these pathways should be differentially expressed following Flovent treatment. To explore this possibility we analyzed the promoter region of differentially expressed genes (q < 0.05) to look for transcription factor binding sites (TFBS) associated with TLR and PPAR pathways. The method *Pscan* was used to find enriched TFBS from the JASPAR and TRANSFAC databases [16]. The top enriched motifs in the JASPAR database were IRF2 and IRF1 (*P* = 4.1E-4 and *P* = 4.1E-3), whereas the top motif in the TRANSFAC database was V$IRSE_01 (*P* = 4.1E-3) (Additional file 4: Table S4). Motifs V$IRF1 (*P* = 0.027), V$IRF2 (*P* = 0.041) and V$IRF7 (*P* = 0.07) were also significantly enriched in the TRANSFAC database. ISRE (Interferon Stimulated Response Element) motifs are involved in the regulation of gene expression in response to interferon type 1 (IFNA-alpha/beta) activation [17]. IRF motifs are the target of Interferon Regulatory Factors (IRFs), which function as transcriptional co-activators of interferon-regulated genes [18,19]. Interestingly, type 1 interferons (alpha and beta) are produced after activation of TLRs (especially *TLR4* and *TLR7*). The TFBS results also showed enrichment of *PPARG*/*RXRA* binding motifs from the JASPAR database (PPARG:::RXRA, *P* = 0.0182) and the TRANSFAC database (V$PPARG_01; *P* = 0.084).

The enrichment motifs determined by *Pscan* provided the possibility that a transcription factor could bind to the promoter of genes. *Cscan *(http://159.149.109.9/cscan/) was used in order to obtain some experimental evidence for binding of transcription factors to the promoter region of the list of differentially expressed genes [20]. *Cscan* compiles ChIP-seq data from published experiments assessing the binding of several transcription factors and epigenetic markers in different cell lines for human and mice. Results from *Cscan* identified GCR as the most significantly enriched transcription factor in six different cell lines subjected to variable doses of a glucocorticoid receptor agonist (Additional file 4: Table S4). STAT1 and RXRA binding were detected at the respective ranks of 10 and 12 (Bonferroni corrected *P*-values: q = 0.0009 and q = 0.0018, respectively). Binding for PPARs or IFR9 could not be assessed due to a lack of available experimental data.

### Interferon regulatory network

Promoter analysis suggested the type 1 interferon pathway as the most important target of GC actions. To further validate this finding we queried the database *Interferome* (http://www.interferome.org), which maintains a curated list of known interferon-regulated genes extracted from studies on which control and interferon-treated samples were analyzed. We found that 20 out of 114 genes in the FDR list (17%) have been previously identified as being regulated by interferons (Additional file 5: Table S5). This results in an associated probability of rejecting the null (no enrichment) hypothesis based on the hypergeometric distribution of *P = 0.0505*, indicating that the FDR list is enriched in interferon-regulated genes. Additional file 5: Table S5 contains the number of datasets in which regulation by a certain type of interferon was found. Type 1 interferons regulated 17 out of 20 genes in a total of 72 datasets, whereas type 2 interferons were the only source of regulation for three genes in a total of 3 datasets. Taken together, these results suggest that GCs target the type 1 interferon pathway in the epithelium of asthmatics.

## Discussion

Classical statistical and functional enrichment analysis of the Flovent/placebo dataset provided little insight into the biological processes associated with the treated asthmatics. Results from the uncorrected tests contained many genes known to be associated with asthma, and some enriched pathways and GO terms linked these genes to inflammatory responses and metabolism of GCs. However, the high number of potential false positives hampered the results and correction for multiple testing eliminated most of these genes from the final list. Genes selected after multiple testing correction (FDR) identified the *SERPINB2* and *FKBP5* genes, which were previously validated [9]. However, the FDR approach failed to identify *CLCA1*, another gene experimentally confirmed [9], although it was significantly altered based on uncorrected *P*-values. This effect could partially be attributed to the reduced statistical power associated with the use of multiple testing correction methods compared to the uncorrected analysis [21]. It has been well demonstrated that the excessive penalty imposed on *P*-values following FDR adjustment increases the number of false negatives [22]. On the other hand, *POSTN*, which was also experimentally validated, had a *P > 0.05* and therefore was not differentially expressed in our analysis. This comparison highlights some of the limitations associated with statistical analyses that rely on a single source of evidence to derive conclusions on biological function, and argues for the need for integrative approaches to data analysis.

Network analysis offers some advantages over classical analysis by being able to incorporate additional information from multiple sources [21,23,24]. We applied a weight-of-evidence approach by using multiple methods that mine different aspects of the cellular processes, which converged to ‘Toll-like receptor signaling pathway’ and ‘PPAR signaling pathway’ as the most relevant pathways related to GC treatment (Figure 4). Toll-like receptors (TLRs) regulate inflammatory responses by inducing the expression of inflammatory cytokines upon binding of viral or bacterial proteins and mediate the signaling pathways that regulate innate and Th2 responses in the epithelium [3,25,26]. Toll-like receptors, including *TLR4* (found in module M6) and *TLR7* (found in module M1) mediate the production of interferons alpha and beta (type 1 interferons) [27]. This important link between TLR and the interferon 1 pathway, not evident from the pathway enrichment analysis, was revealed by the promoter enrichment analysis.

**Figure 5:**
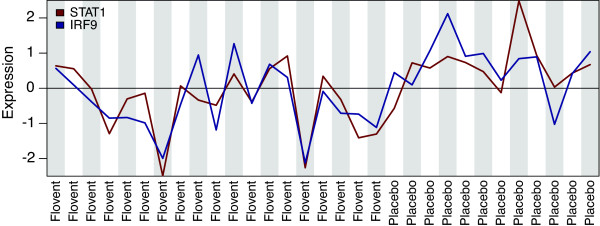
**STAT1 and IRF9 expression profiles.** STAT1 and IRF9 show correlated expression profiles and are downregulated following Flovent treatment.

Type 1 interferons act in an autocrine or paracrine manner and bind to interferon-alpha receptors in the membrane. This activates the Janus kinase and signal transducers and activators of transcription (JAK/STAT) pathway, which regulates the expression of inflammatory genes [17]. Phosphorylation of STAT1 by JAK1 activates STAT1, which forms a heterodimer with STAT2 and recruits IRF9 to form the IFN-stimulated gene factor 3 (ISGF3) transcriptional complex [18,19]. The analysis of the human interactome identified *STAT1* and *IRF9*, which are downregulated (*P < 0.05*) by Flovent (Figure 3). In addition, promoter analysis identified *ISRE* as the top enriched transcription factor motif in the list of differentially expressed genes (q < 0.05). This suggests a mechanism in which GCs regulate the type 1 interferon pathway by downregulating the proteins involved in interferon signal transduction. However, although both *STAT1* and *IRF9* are suggested as differentially expressed (*P < 0.05*) and exhibited correlated expression profiles (r = 0.771, *P* = 3.7E-07*)*, their correlation with treatment is less clear (Figure 5). Interestingly, GCs are known to regulate the type 1 interferon pathway by suppressing the phosphorylation of *STAT1*[28], providing an alternative mechanism of regulation.

STAT1 phosphorylation also mediates the transduction of type 2 interferon (IFN-gamma) signaling by binding to gamma-activated sequence (GAS) elements as an homodimer. Therefore, it is possible that downregulation of *STAT1* and inhibition of phosphorylation of the protein represses type 2 interferon signaling. Indeed, GCs are known to inhibit IFN-gamma signaling by downregulating *STAT1* mRNA and protein expression in PBMCs [29]. Gene expression for type 2 interferon and its receptor (IFN-gamma receptor, IFNGR) were found in the microarray dataset after filtering non-expressed genes (not shown). However, promoter analysis only identified enrichment of ISRE elements, the binding motif for the ISGF3 transcriptional complex, GAS elements were not detected. In addition, interrogation of the interferome database resulted in a list of interferon-regulated genes that were, in the majority of datasets assayed, regulated by type 1 interferons. Consequently, although a role of type 2 interferons cannot be excluded, our findings suggest that type 1 interferons are the target of GCs in the epithelium of asthmatics.

Peroxisome proliferator-activated receptors (PPAR) are lipid-activated transcription factors that regulate the expression of target genes [30]. PPAR-alpha and -gamma modulate allergic inflammation, and agonists are able to reduce levels of inflammatory cytokines [31,32]. PPARs exert their activity by forming a heterodimer with the retinoid receptor *RXRA*, which then binds to co-activator proteins, including *PPARGC1A* (*PGC-1*), to regulate gene expression. Transcriptional co-activators amplify the transcription of nuclear receptor regulated target genes [33]. In particular, PPARGC1A can recruit other co-activator proteins with histone acetyltransferase (HAT) activity that open up the chromatin and enhance the expression of target genes [34]. Consequently, an increase in the expression of *RXRA* and *PPARGC1A* co-activators can intensify the activity of the PPAR pathway, resulting in an increased repression of STAT1 phosphorylation. Both *RXRA* and *PPARGC1A* are present in the BioNet module and are upregulated by Flovent (Figure 3). Promoter analysis also resulted in an enrichment of genes with PPARG:RXRA motifs (*P* ~ 0.01). This suggests that GCs modulates the activity of the PPAR pathway by upregulating co-transcriptional activators. Supporting this evidence, the expression profiles for *RXRA* and *PPARGC1A* are correlated (r = 0.4078, *P* = 0.022) and show a trend of higher levels in Flovent-treated patients (Additional file 3: Figure S8).

Our results link GCs to type 1 interferon and PPAR pathways. There is previous evidence that connects PPARs to interferons, providing an interesting mechanism integrating GC actions. Activation of the PPAR-alpha (*PPARA*) pathway was found to suppress STAT1 phosphorylation in rat glia [35]. However, in our study promoter analysis identified PPARG, but not PPARA (P ~ 0.2), motifs enriched in the list of differentially expressed genes. This lack of significance could be explained by the fact that PPARA motifs are missing in the JASPAR database, which retrieved higher significant results for the complex PPARG:RXRA transcription factor motif. In addition, our search strategy looked for motifs in the 1 kb region upstream of the transcription start site, whereas some transcription factors can bind to more distal locations. Alternatively, as our data suggest, GC action on type 1 interferons may be mediated via a PPAR-gamma-dependent process. For example, it has been shown that PPAR-gamma can repress the type 1 interferon pathway by downregulating the production of INF-beta upon TLR4 activation [36]. Treatment with the PPAR-gamma agonist troglitazone and challenge with LPS and poly(I:C) impaired IRF3 binding to the IFN-beta-promoter. Downregulation of IFN-beta prevented activation of the IFN-beta receptor and subsequent STAT1 phosphorylation and ISRE activation [36]. However, in our dataset type 1 interferons were present, but not differentially expressed (average *P* ~ 0.58 for type 1 interferon genes in the microarray). Interestingly, activation of the PPAR-gamma pathway was previously found to downregulate the expression of IFN-gamma activated genes [37]. These findings highlight the complex nature of PPAR-mediated interferon regulation, which can affect different pathways (type 1 vs. type 2) at multiple regulatory points.

**Figure 6:**
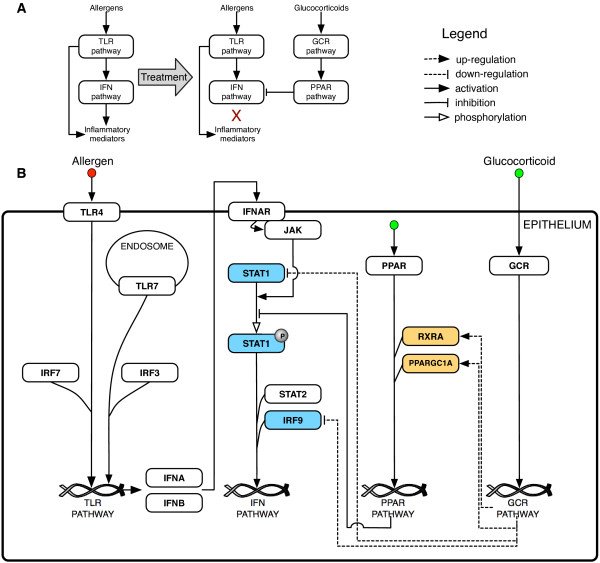
**Model of glucocorticoid (GC) actions in the airways epithelium.** Simplified (**A**) and detailed (**B**) model of PPAR-mediated anti-inflammatory actions of GC in lung epithelial cells. Following activation by allergens, TLRs activate the type 1 interferon pathway. Interferons induce the expression of inflammatory genes mediated by phosphorylated *STAT1* and *IRF9* transcription factors. GC treatment represses the interferon pathway via two potential mechanisms. First, upregulation (orange boxes) of *RXRA* and *PPARGC1A* enhances the activity of the PPAR pathway, which inhibits *STAT1* phosphorylation, repressing interferon signaling. Secondly, down-regulation (blue boxes) of *STAT1* and *IRF9*, reduces the amount of the transcriptional complex responsible for interferon-regulated gene expression. Clear arrowheads indicate phosphorylation. Solid arrowheads indicate activation. A dashed line represents transcriptional regulation (arrow: activation; T: repression).

Our findings provide a link between interferon, PPARs and GCs that suggests a model similar to that presented in Figure 6. In this model, allergens activate the TLR signaling, which in turn activates the production of type 1 interferons. The alpha/beta interferons then bind to interferon receptors (IFNAR1/2) that stimulate the phosphorylation of STAT1 and promote the expression of genes with a number of inflammatory effects. Treatment with GCs upregulates *PPARGC1A* and *RXRA* coactivator molecules, which consequently enhance the PPAR pathway. Activation of the PPAR pathway inhibits phosphorylation of STAT1 and therefore inhibits the interferon pathway. In addition, GCs may repress the interferon pathway by downregulation of *STAT1* and *IRF9* transcription factors. In this model, PPARs could be potential mediators of the anti-inflammatory actions of GCs. Both PPAR-alpha and -gamma inhibit airway inflammation in a murine module of asthma [32]. The use of PPAR-gamma agonists has been shown to evidence improvements in lung function of smokers with asthma (improved FEV_1_; forced expiratory volume in 1 sec), who had previously demonstrated a reduced response to GC treatment [38]. While the mechanism for the observed improvements in lung function was unclear, it was postulated that PPAR-gamma could independently modulate a set of inflammatory genes relative to GCs [39].

A link between GCs and the interferon pathways has been previously reported [5,40-42], and interferons are known to affect symptoms in asthmatics [43-46]. Interferon-alpha has been associated with severe exacerbation of asthma symptoms [43], which provides a simple mechanistic interpretation of the beneficial effects of GC-mediated repression of the interferon pathway. On the other hand, low doses of interferon-alpha have been associated with therapeutic effects in GC-resistant patients [44-46]. It is unclear if these disparate responses are due to differences in interferon dose or different patient phenotype. This clearly highlights the inherent complexity of the underlying regulatory networks and the need of further studies investigating the mechanisms of GCs and their relation with the interferon pathway.

## Conclusions

Multifactorial diseases such as asthma challenge our ability to identify distinct mechanisms involved in disease etiology and pathology. The advent of high-throughput approaches has increased the amount of information that can be extracted from a sample set, but linking together disparate datasets remains an elusive goal. Technologies that summarize the principal disease processes affected (*e.g.,* functional enrichment analysis) assist in characterizing the primary functional features, but often fail to suggest detailed information of disease and treatment action. Accordingly, approaches that incorporate information regarding multiple biological aspects from disparate sources can stimulate the generation of hypotheses that will uncover novel mechanisms. Given that asthma is a heterogeneous inflammatory condition, it is of interest to examine for distinct molecular phenotypes. For example, it has been shown that responsiveness to inhaled GCs correlates with the degree of Th2 inflammation. It is possible that distinct subgroups of asthmatics exist with specific shifts in PPAR/TLR pathways that correlate with GC responsiveness or other molecular phenotypes, such as the IL-13 related Th2 sub-phenotypes previously described for the current data set [47,48]. Further investigations to investigate sub-phenotypical responses in the PPAR/TLR pathways are thus warranted.

With the workflow presented herein, we were able to identify specific pathways as being targets of GCs in the epithelium of asthmatics. Results highlighted the prominent role of interferons in mediating inflammatory processes in asthmatics, and further supported the finding that GCs mediate their activities by repressing the interferon pathway. We found that this repression may be mediated by GC-dependent upregulation of the PPAR pathway. A potential mechanism of direct repression of the interferon pathway by downregulation of key transcription factors is also suggested. If confirmed, these findings will be potentially valuable in the design of new therapies tailored for the GC-resistant asthmatic sub-population.

## Materials and methods

### Microarray data acquisition and preprocessing

The microarray data set was published previously, and is available in the GEO database (accession number GSE4302) [9]. The dataset consisted of 64 Affymetrix HGU133plus2 arrays measuring expression profiles from airway epithelial brush biopsies obtained from 32 asthmatics in a randomized, blinded parallel group study design with sampling at baseline levels and after 1 week treatment with either placebo or fluticasone propionate (Flovent). Observation 55 was identified as an outlier due to poor technical quality of the microarray data, and was excluded from all analyses. Custom CDFs (Chip Description File) from the BrainArray Project were used to map the array probes to 17,788 probe sets corresponding to individual Entrez Gene entries [49]. Arrays were preprocessed with the RMA method from the Bioconductor package *affy*[50,51]. Non-expressed genes were eliminated on the basis of *XIST* expression, which is not expressed in males [52]. The distribution of intensities for *XIST* in male and females was computed (Additional file 3: Figure S9), and the background threshold estimated as the median value of the distribution in males (log2 median intensity = 5.32). Genes were eliminated when the intensity in all arrays was below that of the determined threshold value. The resulting microarray data contained 16,015 remaining genes. Baseline correction was performed by computing the log difference of the intensity values from baseline and post-treated arrays prior to statistical analysis.

### Statistical analysis

Statistical analysis was performed with the Bioconductor package *limma*[53]. A linear model was constructed including drug (*i.e.,* Flovent or placebo), sex and age in the design matrix. P values associated with the comparison Flovent vs. placebo were computed. Multiple testing correction was applied using the method of Benjamini and Hochberg [10].

### Co-transcriptional network

A co-transcriptional network was constructed from the microarray data using the ARACNE method, which uses mutual information (MI) to test statistical dependency between expression profiles [11]. Due to concerns about the effects of low sample numbers in combination with the inherent biological variability upon the statistical dependency, all individuals in the dataset (Flovent-treated and placebo-treated) were included in the co-transcriptional network inference. To reduce the inter-individual variance from the gene expression profiles, expression values were normalized to the baseline values for each individual (*i.e.,* samples collected prior to initialization of treatment regimen). Mutual information was computed using a Gaussian Kernel estimate. Two critical parameters for the ARACNE method are the kernel width and the MI threshold, which are normally estimated using precomputed calibration curves. However, for this project, custom calibration curves were constructed using the MATLAB scripts provided in the original publication [11]. A calibration curve for kernel width was generated (Additional file 3: Figure S10A) and the optimal kernel width was determined to be 0.2179931, which is 5.3% lower than that estimated by ARACNE (0.230167). The calibration curve for the MI threshold was computed (Additional file 3: Figure S10B) and used during network reconstruction. Next, the optimal *P*-value cutoff for network reconstruction was determined by computing networks at different *P*-values over a sufficiently wide range. For each network, the number of nodes, edges and degree distribution was computed (Additional file 3: Figure S11A), and a *P*-value of 1.0E-8 was chosen, which resulted in a power law degree distribution for the network (Additional file 3: Figures S3 and S11B). Finally, in order to assess the effect of sample ordering upon correlation estimations (MI), a bootstrapped network was computed by setting 100 cycles and the consensus network was calculated with a Bonferroni corrected q *< 0.001*[54].

### Protein-protein interaction network

Information about protein-protein interactions (PPI) was obtained from the BioGRID database [15], which contains experimentally validated data from a range of high-throughput and low-throughput techniques. The database version 3.1.70 contained information on 9,935 genes, which formed a network with 50,531 edges. This network contained multiple edges joining the same pair of genes (*i.e.,* the interaction has several sources of evidence), and therefore for module detection the network was simplified by using the *simplify* function in the R package *igraph* (http://igraph.sourceforge.net), to reduce the number of edges per pair of genes to one. The resulting simplified network contained 33,282 edges.

### Module detection

For the co-transcriptional network, detection of clusters of densely connected nodes was performed using the MCODE plugin for the Cytoscape software with default parameters [55]. For the BioGRID PPI network, module detection was performed using the Bioconductor package BioNet [14], which enables the determination of an exact solution to the problem of finding connected sub-graphs with low *P*-values. A Binomial Uniform Mixture (BUM) model was fitted to the distribution of *P*-values, and scores were derived for each network node at a given FDR (q = 0.05). Finally, these scores were used to detect modules using the exact Heinz method [56].

### Functional enrichment analysis

Functional enrichment analysis for KEGG pathways and Gene Ontology (GO) Biological Process (BP) terms (downloaded on 2010/09/07 and 2010/09/04 respectively) was performed using a hypergeometric test as implemented in the Bioconductor package *GOstats*[57]. For GO, a conditional test that considers the dependence structure of the GO terms was performed [58]. The lists of probe sets from the statistical analysis and modules from the co-transcriptional network were converted into lists of Entrez Gene identifiers prior to enrichment analysis. Enrichment results were considered significant at *P < 0.05*.

KEGG pathways were restricted to those involved in biological processes. Consequently, disease pathways were discarded (except the KEGG pathway ‘Asthma’). In addition, pathways that were supported with less than two genes in any module or list of differentially expressed genes were discarded. The final list of KEGG pathways was used to filter modules in the co-transcriptional network. Modules that did not contain any genes annotated in the KEGG pathways that passed the filtering were discarded. Pathways were grouped using a hierarchical clustering with *Pearson* correlation as a distance measure, and the gene counts displayed as a heatmap.

### Common pathways analysis

To link the results from the co-transcriptional and PPI network analyses, common pathways were selected when present simultaneously in the BIONET module and the MCODE module M1 from the co-transcriptional network. To assess the possibility that the selected common pathways appeared by chance in our dataset, we performed simulations with random lists of genes. First, background lists of genes were generated by randomly selecting genes from the total number of genes (universe) used for enrichment analysis. The number of genes in each simulated list was the same as the target list (*i.e.,* the same number of genes as found in the BIONET and MCODE module M1). A KEGG enrichment analysis was then performed for each list as described above, and the common pathways were computed. Finally, the common pathways in the simulation were compared to those obtained with the real dataset and the number of positive hits ***n*** recorded. This process was repeated ***N = 1000*** times and the probability of a pathway appearing by chance was computed as the number of times it appeared as common in the simulated datasets divided by the number of simulations (***P = n / N***).

### Promoter enrichment analysis

Promoter enrichment analysis was performed to identify transcription factor binding motifs enriched in the promoter regions of sets of genes by using the software *Pscan*[16]. Entrez Gene identifiers were converted into REFSEQ ids and these were loaded into the *Pscan* web server. JASPAR and TRANSFAC databases of transcription factor motifs were selected to analyze the region from −950 to +50 bases relative to the transcription start site (TSS). Transcription factor binding assessment was performed to identify experimental evidence (based on published ChIP-seq data) of binding of transcription factors to the promoter region of the differentially expressed genes. Entrez Gene identifiers were converted into REFSEQ ids, which were loaded into the *Cscan* web server [20]. A region between −1,000 upstream of the TSS and the transcribed region of the gene was selected to identify transcription factor binding events.

### Interferon regulated genes

The database Interferome (http://www.interferome.org) was used to identify interferon target genes [59]. This database catalogs genes regulated in microarray and other experiments where comparisons between control and interferon treated samples were performed. Entrez Gene identifiers were converted into EnsEMBL gene identifiers and loaded into the Interferome web server. Enrichment of interferon-regulated genes was computed using the hypergeometric test implemented in the R software. The parameters were set with 20,900 as total number of genes in the human genome (based on data from the EnsEMBL database release 64) and 2,000 as the number of interferon-regulated genes (as stated in the Interferome web site). Since the number of interferon-regulated genes is an estimate, the enrichment calculation is only approximate.

## Competing interest

The authors declare no competing interests.

## Authors’ contributions

DD helped to design the study, performed the analyses and wrote the manuscript. SG provided computational resources for network generation and assisted in development of the methods for pathway enrichment analysis. JVF helped design the study and provided the initial microarray dataset. DJE helped design the study, provided the initial microarray dataset, and critically revised the manuscript. PGW helped design the study, provided the initial microarray dataset, provided clinical input, and helped critically revise the manuscript. ÅMW conceived of the study and assisted in writing the manuscript. CEW conceived of the study, assisted in the analyses, and assisted in writing the manuscript. All authors have read and approved the final manuscript.

## Pre-publication history

The pre-publication history for this paper can be accessed here:

http://www.biomedcentral.com/1755-8794/5/27/prepub

## Supplementary Material

Additional file 1 **Table S1.** Stat_analysis_results.xlsx (Microsoft Excel XLSX) Lists of genes found significantly changed in the statistical analysis. Click here for file

Additional file 2 **Table S2.** Func_enrich_results.xlsx (Microsoft Excel XLSX) Results for KEGG pathways and GO terms functional enrichment analysis. Click here for file

Additional file 3 Supp_material.pdf (Adobe PDF) Supplementary information and figures for the main manuscript [11,52,54]. Click here for file

Additional file 4 **Table S4.** Promoter analysis.xlsx (Microsoft Excel XLSX) Results from *Pscan* promoter enrichment analysis with JASPAR and TRANSFAC, and results from *Cscan* ChIP-seq analysis. Click here for file

Additional file 5 **Table S5.** Interferon_regulated_genes.xlsx (Microsoft Excel XLSX) List of known interferon-regulated genes from the FDR set. Click here for file

## References

[B1] Fanta CH Asthma N Eng J Med 2009 360 10 1002 1014 10.1056/NEJMra0804579 19264689

[B2] Global Initiative for Asthma (GINA) Global strategy for asthma management and prevention 2010 Global Initiative for Asthma (GINA), Bethesda (MD)

[B3] Bulek K Swaidani S Aronica M Li X Epithelium: the interplay between innate and Th2 immunity Immunol Cell Biol 2010 88 3 257 268 10.1038/icb.2009.113 20065993

[B4] Hammad H Lambrecht BN Dendritic cells and epithelial cells: linking innate and adaptive immunity in asthma Nat Rev Immunol 2008 8 3 193 204 10.1038/nri2275 18301423

[B5] Stellato C Glucocorticoid actions on airway epithelial responses in immunity: functional outcomes and molecular targets J Allergy Clin Immunol 2007 120 6 1247 1263 quiz 1264–1245 10.1016/j.jaci.2007.10.041 18073120

[B6] Barnes PJ Inhaled glucocorticoids for asthma N Eng J Med 1995 332 13 868 875 10.1056/NEJM199503303321307 7870143

[B7] Barnes PJ Glucocorticosteroids: current and future directions Br J Pharmacol 2010 120 2-3 76 85 10.1111/j.1476-5381.2010.01199.x PMC3085866 21198556

[B8] Barnes PJ Adcock IM Glucocorticoid resistance in inflammatory diseases Lancet 2009 373 9678 1905 1917 10.1016/S0140-6736(09)60326-3 19482216

[B9] Woodruff PG Boushey HA Dolganov GM Barker CS Yang YH Donnelly S Ellwanger A Sidhu SS Dao-Pick TP Pantoja C Genome-wide profiling identifies epithelial cell genes associated with asthma and with treatment response to corticosteroids Proc Natl Acad Sci USA 2007 104 40 15858 15863 10.1073/pnas.0707413104 17898169 PMC2000427

[B10] Benjamini Y Hochberg Y Controlling the False Discovery Rate: A Practical and Powerful Approach to Multiple Testing Journal of the Royal Statistical Society Series B (Methodological) 1995 57 289 300

[B11] Margolin AA Nemenman I Basso K Wiggins C Stolovitzky G Dalla Favera R Califano A ARACNE: an algorithm for the reconstruction of gene regulatory networks in a mammalian cellular context BMC Bioinformatics 2006 7 1 S7 10.1186/1471-2105-7-S1-S7 16723010 PMC1810318

[B12] Shi Z Derow CK Zhang B Co-expression module analysis reveals biological processes, genomic gain, and regulatory mechanisms associated with breast cancer progression BMC Syst Biol 2010 4 74 10.1186/1752-0509-4-74 20507583 PMC2902438

[B13] Ideker T Ozier O Schwikowski B Siegel AF Discovering regulatory and signalling circuits in molecular interaction networks Bioinformatics 2002 18 Suppl 1 S233 S240 10.1093/bioinformatics/18.suppl_1.S233 12169552

[B14] Beisser D Klau GW Dandekar T Muller T Dittrich MT BioNet: an R-Package for the functional analysis of biological networks Bioinformatics 2010 26 8 1129 1130 10.1093/bioinformatics/btq089 20189939

[B15] Stark C Breitkreutz BJ Reguly T Boucher L Breitkreutz A Tyers M BioGRID: a general repository for interaction datasets Nucleic Acids Res 2006 34 D535 D539 Database issue 10.1093/nar/gkj109 16381927 PMC1347471

[B16] Zambelli F Pesole G Pavesi G Pscan: finding over-represented transcription factor binding site motifs in sequences from co-regulated or co-expressed genes Nucleic Acids Res 2009 37 W247 W252 Web Server issue 10.1093/nar/gkp464 19487240 PMC2703934

[B17] Darnell JE Kerr IM Stark GR Jak-STAT pathways and transcriptional activation in response to IFNs and other extracellular signaling proteins Science 1994 264 5164 1415 1421 10.1126/science.8197455 8197455

[B18] Lawrence T Natoli G Transcriptional regulation of macrophage polarization: enabling diversity with identity Nat Rev Immunol 2011 11 11 750 761 10.1038/nri3088 22025054

[B19] Hervas-Stubbs S Perez-Gracia JL Rouzaut A Sanmamed MF Le Bon A Melero I Direct effects of type I interferons on cells of the immune system Clin Cancer Res 2011 17 9 2619 2627 10.1158/1078-0432.CCR-10-1114 21372217

[B20] Zambelli F Pavesi G Cscan: Finding Common Regulators In A Set Of Genes Using Genome-Wide Chip-Seq Data Next Generation Sequencing Workshop 2011 Bari, Italy October 12–14 2011

[B21] Diez D Wheelock AM Goto S Haeggstrom JZ Paulsson-Berne G Hansson GK Hedin U Gabrielsen A Wheelock CE The use of network analyses for elucidating mechanisms in cardiovascular disease Mol Biosyst 2010 6 2 289 304 10.1039/b912078e 20094647

[B22] Ioannidis JP Tarone R McLaughlin JK The false-positive to false-negative ratio in epidemiologic studies Epidemiology 2011 22 4 450 456 10.1097/EDE.0b013e31821b506e 21490505

[B23] Barabasi AL Gulbahce N Loscalzo J Network medicine: a network-based approach to human disease Nat Rev Genet 2011 12 1 56 68 10.1038/nrg2918 21164525 PMC3140052

[B24] Lee DS Park J Kay KA Christakis NA Oltvai ZN Barabasi AL The implications of human metabolic network topology for disease comorbidity Proc Natl Acad Sci USA 2008 105 29 9880 9885 10.1073/pnas.0802208105 18599447 PMC2481357

[B25] Uematsu S Akira S Toll-like receptors and innate immunity J Mol Med (Berl) 2006 84 9 712 725 10.1007/s00109-006-0084-y 16924467

[B26] Lambrecht BN Hammad H The role of dendritic and epithelial cells as master regulators of allergic airway inflammation Lancet 2010 376 9743 835 843 10.1016/S0140-6736(10)61226-3 20816550

[B27] Baccala R Hoebe K Kono DH Beutler B Theofilopoulos AN TLR-dependent and TLR-independent pathways of type I interferon induction in systemic autoimmunity Nat Med 2007 13 5 543 551 10.1038/nm1590 17479100

[B28] Bhattacharyya S Zhao Y Kay TW Muglia LJ Glucocorticoids target suppressor of cytokine signaling 1 (SOCS1) and type 1 interferons to regulate Toll-like receptor-induced STAT1 activation Proc Natl Acad Sci USA 2011 108 23 9554 9559 10.1073/pnas.1017296108 21606371 PMC3111275

[B29] Hu X Li WP Meng C Ivashkiv LB Inhibition of IFN-gamma signaling by glucocorticoids J Immunol 2003 170 9 4833 4839 12707366 10.4049/jimmunol.170.9.4833

[B30] Kelly DP The pleiotropic nature of the vascular PPAR gene regulatory pathway Circ Res 2001 89 11 935 937 11717147

[B31] Lee KS Park SJ Hwang PH Yi HK Song CH Chai OH Kim JS Lee MK Lee YC PPAR-gamma modulates allergic inflammation through up-regulation of PTEN FASEB J 2005 19 8 1033 1035 15788448 10.1096/fj.04-3309fje

[B32] Trifilieff A Bench A Hanley M Bayley D Campbell E Whittaker P PPAR-alpha and -gamma but not -delta agonists inhibit airway inflammation in a murine model of asthma: in vitro evidence for an NF-kappaB-independent effect Br J Pharmacol 2003 139 1 163 171 10.1038/sj.bjp.0705232 12746235 PMC1573830

[B33] Wolf IM Heitzer MD Grubisha M DeFranco DB Coactivators and nuclear receptor transactivation J Cell Biochem 2008 104 5 1580 1586 10.1002/jcb.21755 18393355

[B34] Liu C Lin JD PGC-1 coactivators in the control of energy metabolism Acta Biochim Biophys Sin 2011 43 4 248 257 10.1093/abbs/gmr007 21325336 PMC3063079

[B35] Lee JH Joe EH Jou I PPAR-alpha activators suppress STAT1 inflammatory signaling in lipopolysaccharide-activated rat glia Neuroreport 2005 16 8 829 833 10.1097/00001756-200505310-00010 15891579

[B36] Zhao W Wang L Zhang M Wang P Zhang L Yuan C Qi J Qiao Y Kuo PC Gao C Peroxisome proliferator-activated receptor gamma negatively regulates IFN-beta production in Toll-like receptor (TLR) 3- and TLR4-stimulated macrophages by preventing interferon regulatory factor 3 binding to the IFN-beta promoter J Biol Chem 2011 286 7 5519 5528 10.1074/jbc.M110.149823 21148557 PMC3037665

[B37] Marx N Mach F Sauty A Leung JH Sarafi MN Ransohoff RM Libby P Plutzky J Luster AD Peroxisome proliferator-activated receptor-gamma activators inhibit IFN-gamma-induced expression of the T cell-active CXC chemokines IP-10, Mig, and I-TAC in human endothelial cells J Immunol 2000 164 12 6503 6508 10843708 10.4049/jimmunol.164.12.6503 PMC4231715

[B38] Spears M Donnelly I Jolly L Brannigan M Ito K McSharry C Lafferty J Chaudhuri R Braganza G Bareille P Bronchodilatory effect of the PPAR-gamma agonist rosiglitazone in smokers with asthma Clin Pharmacol Ther 2009 86 1 49 53 10.1038/clpt.2009.41 19357642

[B39] Ogawa S Lozach J Benner C Pascual G Tangirala RK Westin S Hoffmann A Subramaniam S David M Rosenfeld MG Molecular determinants of crosstalk between nuclear receptors and toll-like receptors Cell 2005 122 5 707 721 10.1016/j.cell.2005.06.029 16143103 PMC1430687

[B40] Stojadinovic O Lee B Vouthounis C Vukelic S Pastar I Blumenberg M Brem H Tomic-Canic M Novel genomic effects of glucocorticoids in epidermal keratinocytes: inhibition of apoptosis, interferon-gamma pathway, and wound healing along with promotion of terminal differentiation J Biol Chem 2007 282 6 4021 4034 17095510 10.1074/jbc.M606262200

[B41] Tliba O Cidlowski JA Amrani Y CD38 expression is insensitive to steroid action in cells treated with tumor necrosis factor-alpha and interferon-gamma by a mechanism involving the up-regulation of the glucocorticoid receptor beta isoform Mol Pharmacol 2006 69 2 588 596 16291871 10.1124/mol.105.019679

[B42] Tliba O Damera G Banerjee A Gu S Baidouri H Keslacy S Amrani Y Cytokines induce an early steroid resistance in airway smooth muscle cells: novel role of interferon regulatory factor-1 Am J Respir Cell Mol Biol 2008 38 4 463 472 10.1165/rcmb.2007-0226OC 17947510 PMC2274949

[B43] Bini EJ Weinshel EH Severe exacerbation of asthma: a new side effect of interferon-alpha in patients with asthma and chronic hepatitis C Mayo Clin Proc 1999 74 4 367 370 10.4065/74.4.367 10221466

[B44] Gratzl S Palca A Schmitz M Simon HU Treatment with IFN-alpha in corticosteroid-unresponsive asthma J Allergy Clin Immunol 2000 105 5 1035 1036 10.1067/mai.2000.105317 10808188

[B45] Mouthon L Guillevin L Interferon-alpha in corticosteroid-resistant asthma and Churg-Strauss syndrome Allergy 2003 58 12 1244 1246 10.1046/j.1398-9995.2003.00347.x 14616097

[B46] Simon HU Seelbach H Ehmann R Schmitz M Clinical and immunological effects of low-dose IFN-alpha treatment in patients with corticosteroid-resistant asthma Allergy 2003 58 12 1250 1255 10.1046/j.1398-9995.2003.00424.x 14616099

[B47] Woodruff PG Modrek B Choy DF Jia G Abbas AR Ellwanger A Koth LL Arron JR Fahy JV T-helper type 2-driven inflammation defines major subphenotypes of asthma Am J Respir Crit Care Med 2009 180 5 388 395 10.1164/rccm.200903-0392OC 19483109 PMC2742757

[B48] Choy DF Modrek B Abbas AR Kummerfeld S Clark HF Wu LC Fedorowicz G Modrusan Z Fahy JV Woodruff PG Gene expression patterns of Th2 inflammation and intercellular communication in asthmatic airways J Immunol 2011 186 3 1861 1869 10.4049/jimmunol.1002568 21187436 PMC3981556

[B49] Dai M Wang P Boyd AD Kostov G Athey B Jones EG Bunney WE Myers RM Speed TP Akil H Evolving gene/transcript definitions significantly alter the interpretation of GeneChip data Nucleic Acids Res 2005 33 20 e175 10.1093/nar/gni179 16284200 PMC1283542

[B50] Gautier L Cope L Bolstad BM Irizarry RA affy–analysis of Affymetrix GeneChip data at the probe level Bioinformatics 2004 20 3 307 315 10.1093/bioinformatics/btg405 14960456

[B51] Gentleman RC Carey VJ Bates DM Bolstad B Dettling M Dudoit S Ellis B Gautier L Ge Y Gentry J Bioconductor: open software development for computational biology and bioinformatics Genome Biol 2004 5 10 R80 10.1186/gb-2004-5-10-r80 15461798 PMC545600

[B52] Brown CJ Hendrich BD Rupert JL Lafreniere RG Xing Y Lawrence J Willard HF The human XIST gene: analysis of a 17 kb inactive X-specific RNA that contains conserved repeats and is highly localized within the nucleus Cell 1992 71 3 527 542 10.1016/0092-8674(92)90520-M 1423611

[B53] Smyth GK Gentleman R, Carey V, Dudoit S, Irizarry R, Huber W Limma: linear models for microarray data Bioinformatics and Computational Biology Solutions using R and Bioconductor 2005 Springer, New York 397 420

[B54] Bonferroni CE Il calcolo delle assicurazioni su gruppi di teste Studi in Onore del Professore Salvatore Ortu Carboni 1935 13 60

[B55] Bader GD Hogue CW An automated method for finding molecular complexes in large protein interaction networks BMC Bioinformatics 2003 4 2 10.1186/1471-2105-4-2 12525261 PMC149346

[B56] Dittrich MT Klau GW Rosenwald A Dandekar T Muller T Identifying functional modules in protein-protein interaction networks: an integrated exact approach Bioinformatics 2008 24 13 i223 i231 10.1093/bioinformatics/btn161 18586718 PMC2718639

[B57] Falcon S Gentleman R Using GOstats to test gene lists for GO term association Bioinformatics 2007 23 2 257 258 10.1093/bioinformatics/btl567 17098774

[B58] Alexa A Rahnenfuhrer J Lengauer T Improved scoring of functional groups from gene expression data by decorrelating GO graph structure Bioinformatics 2006 22 13 1600 1607 10.1093/bioinformatics/btl140 16606683

[B59] Samarajiwa SA Forster S Auchettl K Hertzog PJ INTERFEROME: the database of interferon regulated genes Nucleic Acids Res 2009 37 D852 D857 Database issue 10.1093/nar/gkn732 18996892 PMC2686605

