# Supplementary material for: Network analysis identifies a putative role for the PPAR and type 1 interferon pathways in glucocorticoid actions in asthmatics
Source: BMC Med Genomics. 2012 Jun 19;5:27. doi: 10.1186/1755-8794-5-27 (PMC3408345; doi:10.1186/1755-8794-5-27)
Supplement: Additional file 3 — Supp_material.pdf (Adobe PDF) Supplementary information and figures for the main manuscript [11,52,54]. [file 1755-8794-5-27-S3.pdf]

Network analysis identifies a putative role for the PPAR and type 1 interferon pathways in glucocorticoid actions in asthmatics

Supplementary material

Diego Diez[1†], Susumu Goto[1], John V. Fahy[2,4], David J. Erle[2,3,4], Prescott G. Woodruff[2,4], Åsa M. Wheelock[5*], Craig E. Wheelock[6*]

[1] Bioinformatics Center, Institute for Chemical Research, Kyoto University, Uji, Kyoto, 611-0011, Japan

[2] Division of Pulmonary and Critical Care Medicine, Department of Medicine, University of California San Francisco, USA

[3] Lung Biology Center, University of California San Francisco, USA

[4] Cardiovascular Research Institute, University of California San Francisco, USA

[5] Respiratory Medicine Unit, Department of Medicine, Karolinska Institutet, Stockholm, Sweden

[6] Department of Medical Biochemistry and Biophysics, Division of Physiological Chemistry II, Karolinska Institutet, Stockholm, Sweden

†**Current address:** Laboratory of Bioinformatics and Genomics, World Premier International Immunology Frontier Research Center, Osaka University, Osaka 565-0871, Japan

*** Corresponding author:**
Craig E. Wheelock: craig.wheelock@ki.se

Åsa M. Wheelock: asa.wheelock@ki.se

Construction of the co-transcriptional network

A co-transcriptional network was constructed from the microarray data using the ARACNE method, which uses mutual information (MI) to test statistical dependency between expression profiles [2]. Mutual information was computed using a Gaussian Kernel estimate. Two critical parameters for the ARACNE method are the kernel width and the MI threshold, which is the minimum MI value to be considered statistically significant. Both parameters are normally estimated using precomputed calibration curves that were generated for a different microarray platform. To avoid bias due to the platform differences and ensure the best parameters for our dataset are used, custom calibration curves were constructed using the MATLAB scripts provided in the original publication [2]. A calibration curve for kernel width was generated (Figure S11A) and the optimal kernel width was determined to be 0.2179931, which is 5.3% lower than that estimated by ARACNE (0.230167). The calibration curve for the MI threshold was computed (Figure S11B) and used during network reconstruction. Next, the optimal p-value cutoff for network reconstruction was determined by computing networks at different p-values over a sufficiently wide range. For each network, the number of nodes, edges and degree distribution was computed, and a p-value of 10^{-8} was chosen, which resulted in a power law degree distribution for the network (Figure S12). Finally, in order to assess the effect of sample ordering upon correlation estimations (MI), a bootstrapped network was computed by setting 100 cycles and the consensus network was calculated with a Bonferroni corrected $q < 0.001$ [3]. The inferred network contained 7,676 nodes and 49,499 edges (Figure S1), and the degree distribution followed a power-law with a gamma parameter equal to 1.69 (Figure S2).

Module detection in the co-transcriptional network

Clustering of the co-transcriptional network was performed using MCODE. Several modules were selected based on statistical, functional and relevance to asthma, inflammation, epithelium and metabolism of steroids. Figure S1 shows the location of some MCODE modules (M1, M5, M6, M9) and the BioNet module in the co-transcriptional network, highlighting the dependency between modules M1 and M6. Figure S4 shows the expression profiles of modules M1, M5 (for up and down-regulated genes) and M6, indicating that M1 and M6 genes have highly correlated expression profiles. Figures S3, S5 and S6 show modules M5, M6 and M9 with the selected KEGG pathways and top-ten ranked GO biological processes detected by enrichment analysis.

Filtering of non-expressed genes

Non-expressed genes were eliminated on the basis of XIST expression, which is not expressed in males [1]. The distribution of intensities for XIST in male and females was computed (Figure S10), and the background threshold estimated as the median value of the distribution in males (5.32). Genes were filtered when the intensity in all arrays was below that of the determined threshold value.

Expression profiles for selected genes

The most relevant pathways selected in our analysis were Toll-like receptor signaling pathway and PPAR signaling. Four genes associated with these pathways were identified in the BioNet module. Figure S8 show the expression profiles of STAT1 and IRF9, which mediate signaling by TLR7 and TLR4, and are involved in the production of type 1 interferons. Both genes have correlated profiles that are also somewhat correlated with treatment. Figure S9 shows RXRA and PPARGC1A, which mediate signaling by PPARs, and are involved in the inhibition of inflammatory responses. Both profiles are correlated and correlated as well with treatment.

Figure S1. Summary of functional enrichment analysis. Heatmap displaying the gene count distribution of KEGG pathways enriched in the gene lists from the different analyses. RAW = the list of genes based upon *P*-values that were not corrected for multiple testing ($P<0.05$). FDR = the list of genes following multiple testing correction ($q<0.05$). BIONET = the module resulting from the BioNet analysis of the PPI network. M1 = module that had the highest score following MCODE analysis of the co-transcriptional network.

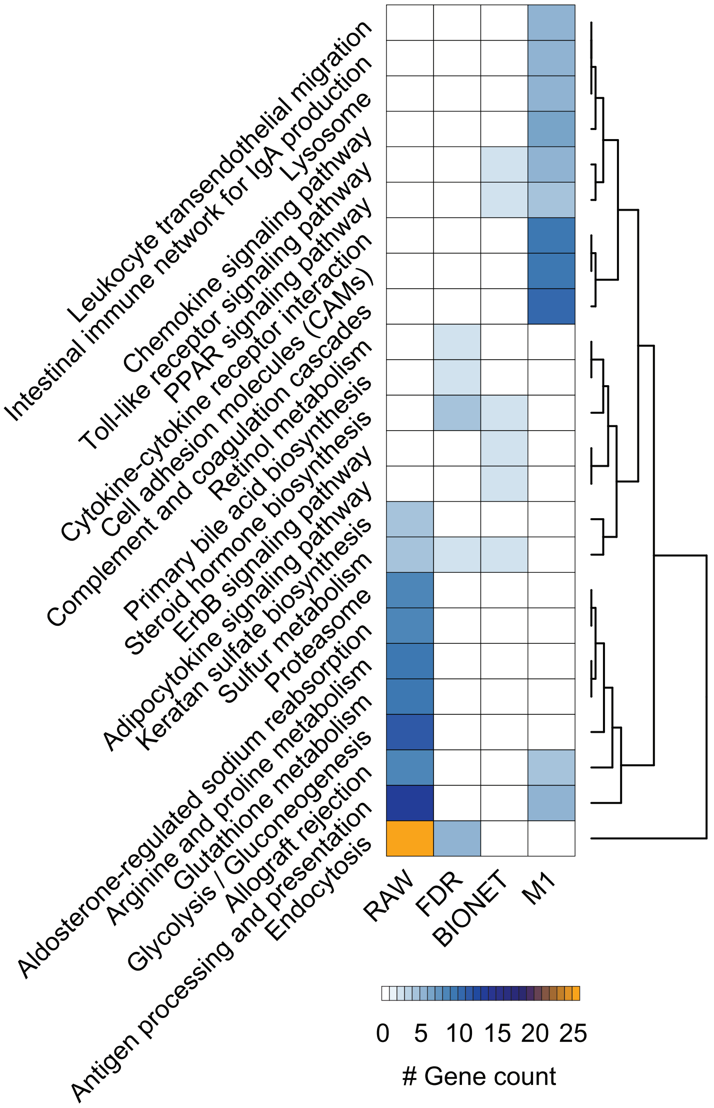

Figure S2. Co-transcriptional network. ARACNE-based network with selected modules visualized. Color key: M1 (yellow), M5 (cyan), M6 (green), M9 (red) and BioNet (pink). The functional relation between MCODE modules M1 and M6 is highlighted by the close proximity of the modules in the whole co-transcriptional network.

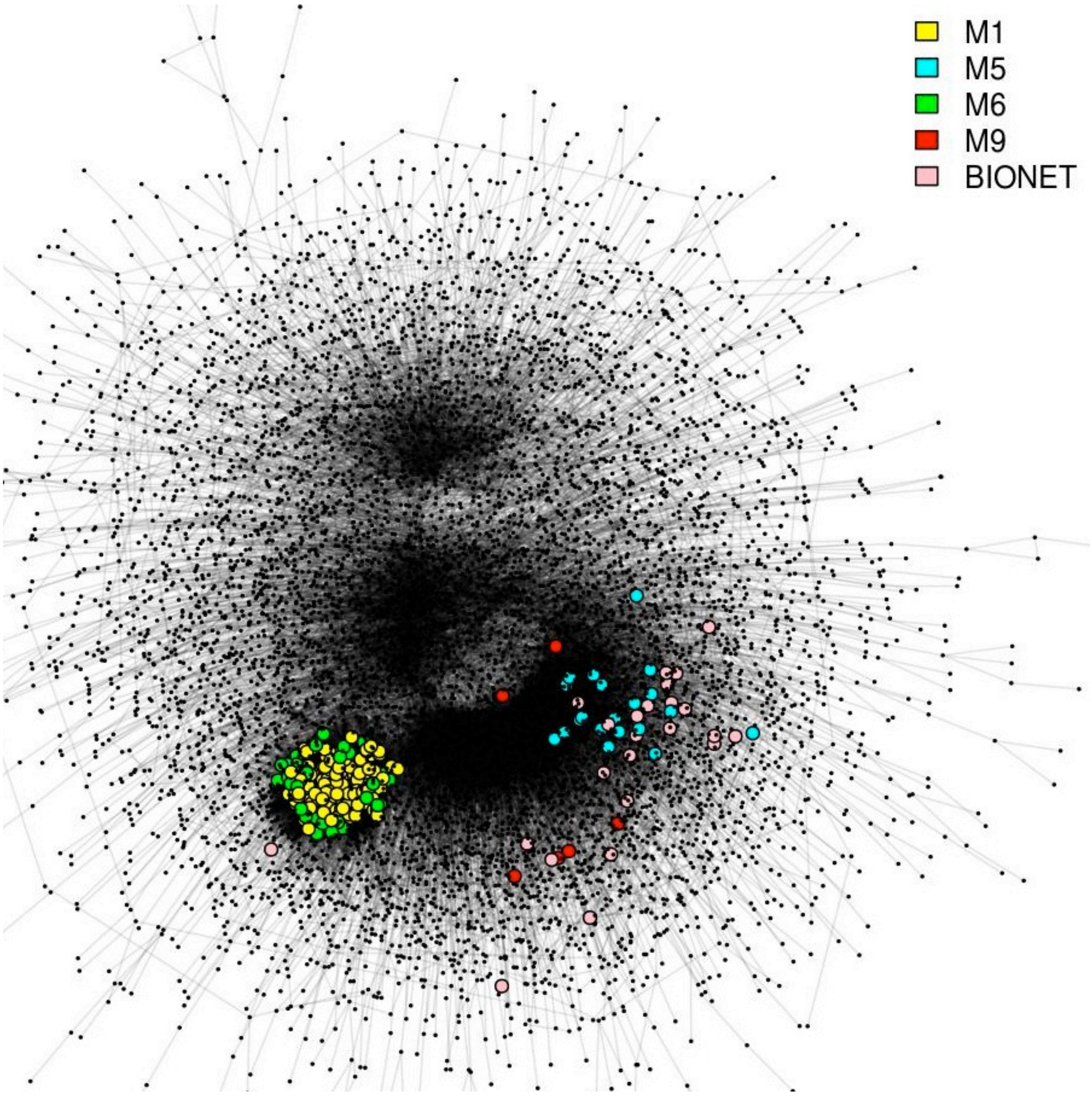

Figure S3. Co-transcriptional network degree distribution. The degree distribution for the ARACNE inferred co-transcriptional network follows a power law with gamma = -1.69.

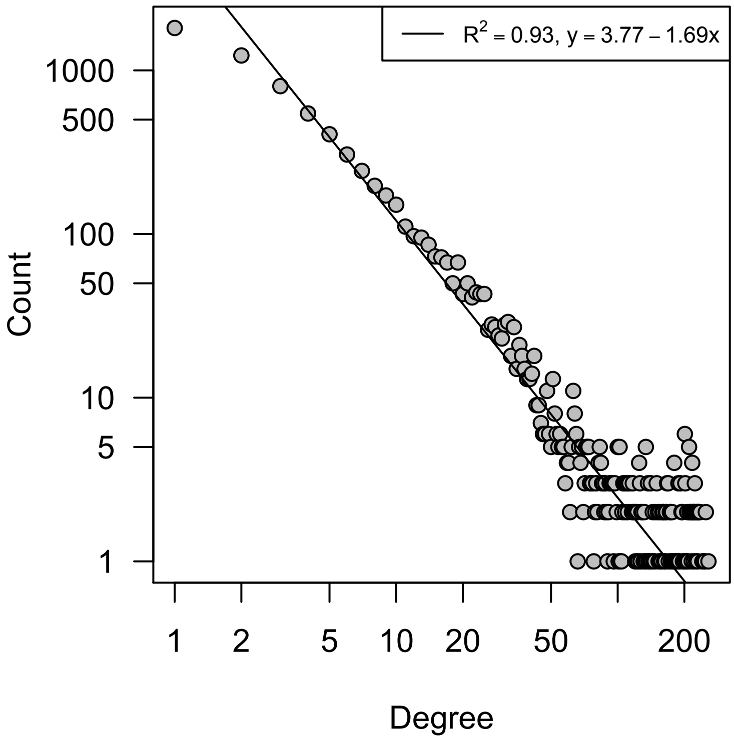

Figure S4. MCODE module M5. All genes in this module are differentially expressed with q<0.05. Therefore, this module was discarded from the common pathways approach. Enrichment analysis returned 'Steroid hormone biosynthesis' as the most significantly enriched pathway, overlapping with the most significant pathway enriched in the FDR list.

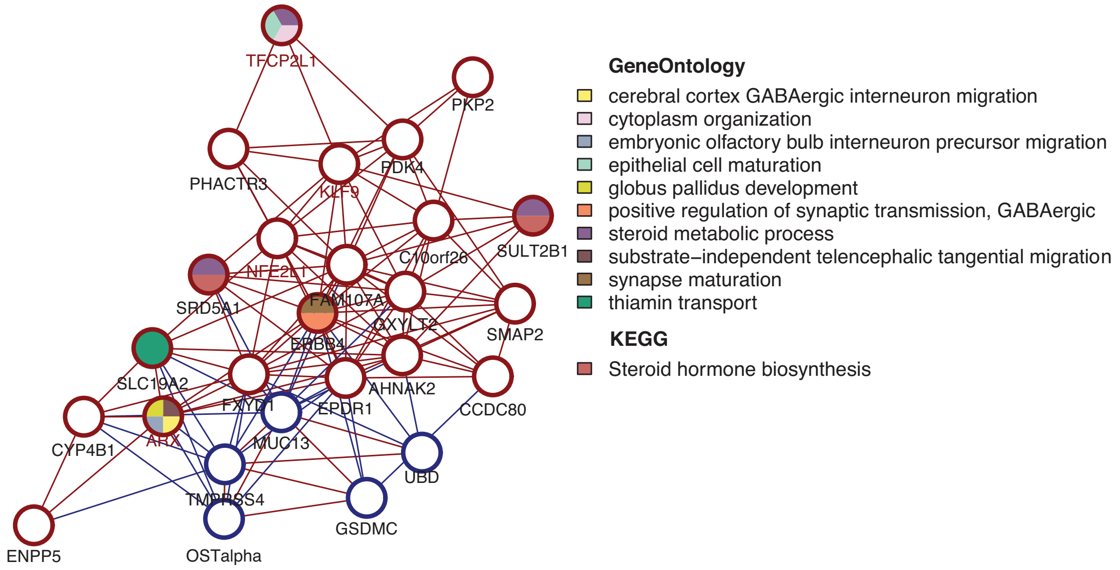

Figure S5. Expression profiles of selected MCODE modules. Profile plots of genes in modules M1 (darkgreen), M6 (black) and BioNet (darkred: up-regulated in flovent; darkblue: down-regulated in flovent). Functional relation between M1 and M6 is evidenced by they correlated profiles. Additionally, their independence from drug variable is also revealed. The genes in module M5, on the other hand, show correlation with treatment.

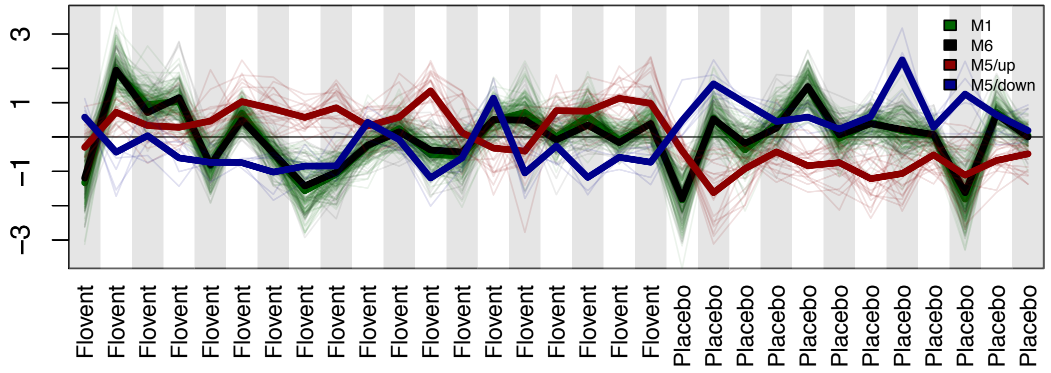

Figure S6. MCODE module M6.

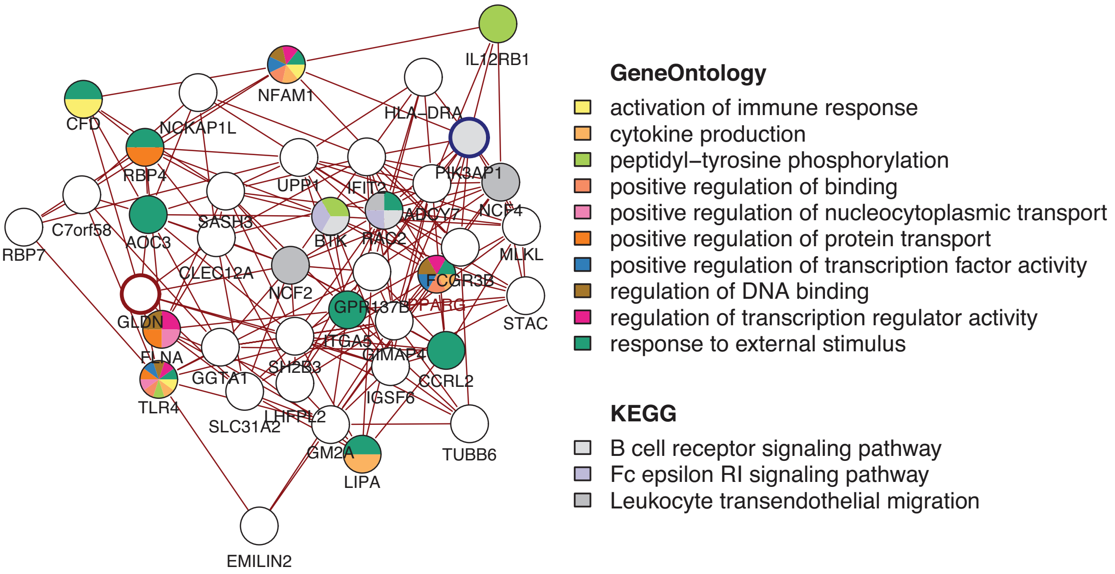

Figure S7. MCODE module M9.

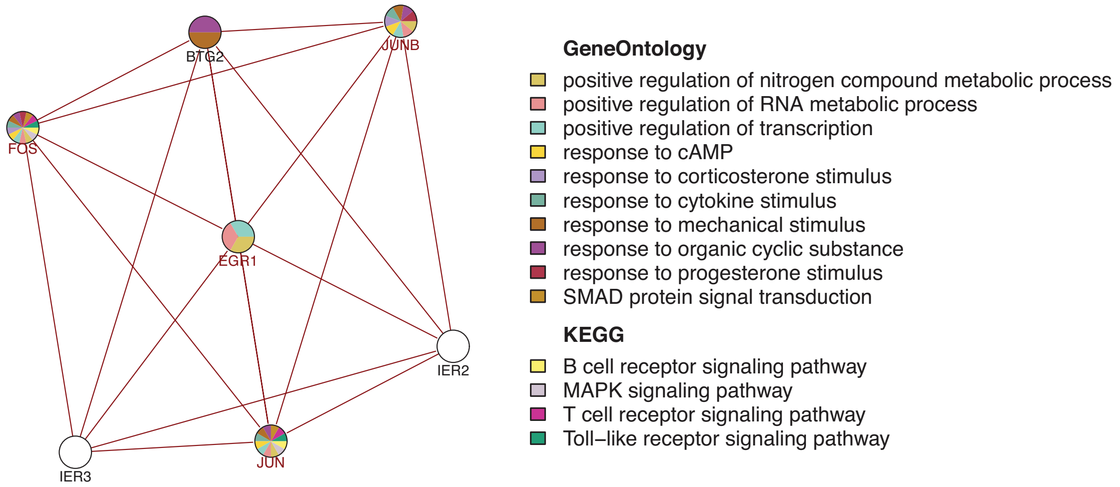

Figure S8. RXRA and PPARGC1A expression profiles. RXRA and PPARGC1A show correlated expression profiles and are upregulated in flovent.

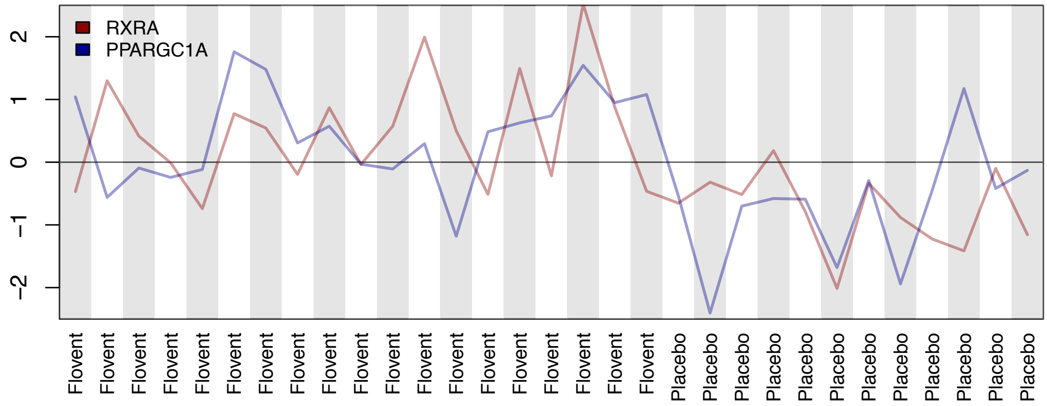

Figure S9. Filtering of non-expressed genes was performed based on the expression of the gene XIST, which is missing in males and provides a lower intensity threshold.

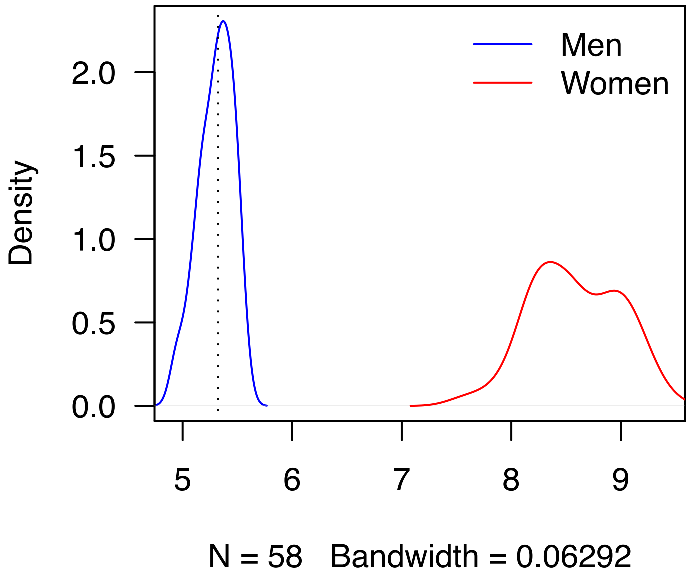

Figure S10. A) Calibration curve for the kernel width computed for different samples sizes. B) Calibration curve for optimal MI threshold computation. Each measurement is performed in triplicate at each sample size.

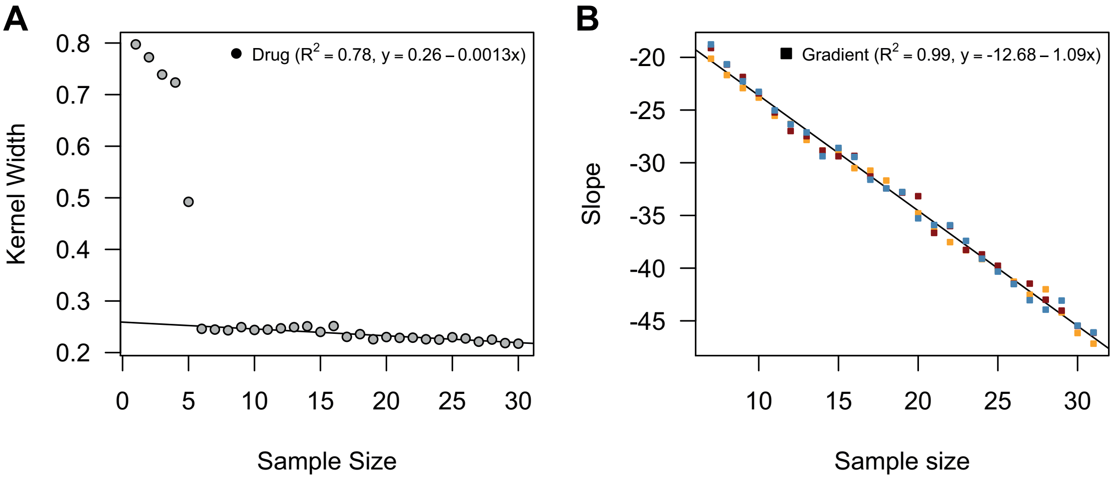

Figure S11. A) Number of nodes and edges vs. P value profile for the drug co-transcriptional network. B) Degree distribution for networks constructed at different P value cutoffs. These plots show that at high P values the networks behave as random networks, with a number of nodes equal to the total number of genes in the array, and a large number of edges. As the P value decreases, the networks reduce the number of edges, and eventually the number of nodes (left). At the same time, the behavior of the networks changes from random to a scale free (right; blue networks). Finally, the number of nodes and edges is too low, and the network looses its structures.

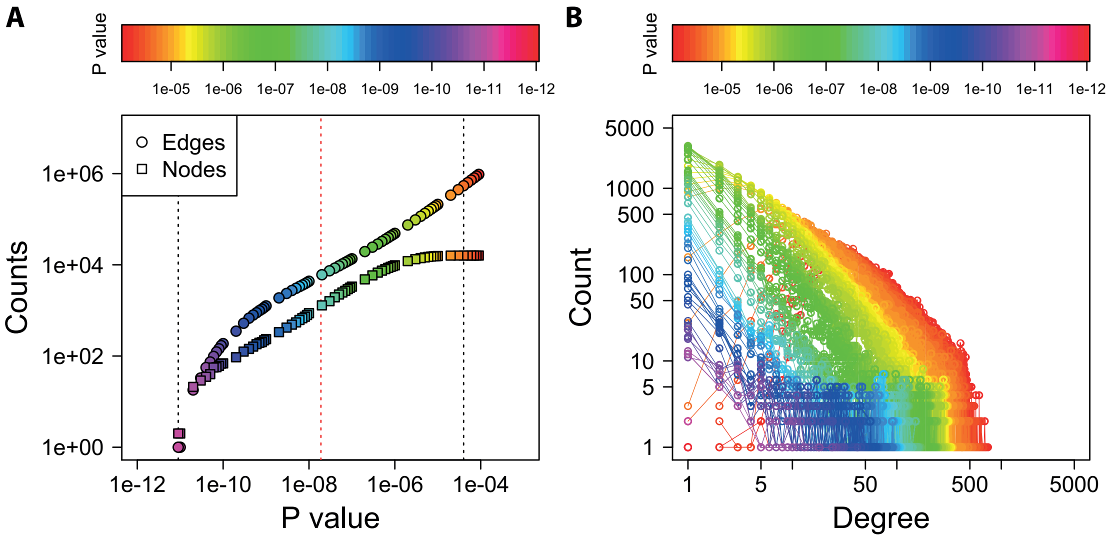

References

1. Brown CJ, Hendrich BD, Rupert JL, Lafreniere RG, Xing Y, Lawrence J, Willard HF: **The human XIST gene: analysis of a 17 kb inactive X-specific RNA that contains conserved repeats and is highly localized within the nucleus.** *Cell* 1992, **71:**527-542.
2. Margolin AA, Nemenman I, Basso K, Wiggins C, Stolovitzky G, Dalla Favera R, Califano A: **ARACNE: an algorithm for the reconstruction of gene regulatory networks in a mammalian cellular context.** *BMC Bioinformatics* 2006, **7 Suppl 1:**S7.
3. Bonferroni CE: **Il calcolo delle assicurazioni su gruppi di teste.** *Studi in Onore del Professore Salvatore Ortu Carboni* 1935**:**13-60.
